# Prevalence of rare visceral aneurysms

**DOI:** 10.1590/1677-5449.202500412

**Published:** 2025-07-07

**Authors:** Jamile Gomes Pereira de Barros, Luana Vieira de Medeiros Santana, Maria Clara Gomes Pontes, Letícia Lemos Belo, Bárbara Rocha Rodrigues Cox Coelho, Lucas Mendes Reis de Moura, Pedro Pereira Tenório

**Affiliations:** 1 Universidade Federal do Vale do São Francisco – UNIVASF, Paulo Afonso, BA, Brasil.

Dear Editor,

We read with attention the article entitled “Prevalence of rare visceral aneurysms on 92,833 CAT scans (2005-2021)”, which we consider an essential contribution to the academic community, and we congratulate the authors on conducting the study. However, certain structural and interpretative weaknesses merit attention.

Analyzing the study aims, we observed that the only objective outlined by the authors was to evaluate the prevalence of rare visceral aneurysms. The article appears to aim to increase use of computed tomography angiography, which could be considered iatrogeny in the absence of any plausible medical justification.^1,2^ As such, it is important to analyze how conducting computed tomography angiographies to determine prevalence rates of visceral aneurysms would change clinical outcomes for patients, in order to avoid exposing them to additional diagnostic tests which may be irrelevant to their health.^1-3^

The authors state that 158 patients with visceral aneurysms had a total of 163 aneurysms, but, at another point in the article, mention 163 patients with visceral aneurysms, which hampers comprehension of the information and statistical data presented.

Finally, we congratulate the journal and the esteemed authors for producing and publishing this paper, given its relevance to the scientific community, considering the small number of studies dealing with the subject.
