# Supplementary material for: Prevalence of rare visceral aneurysms
Source: J Vasc Bras. 2025 Jul 7;24:e20250041. doi: 10.1590/1677-5449.202500412 (PMC12258811; doi:10.1590/1677-5449.202500412)
Supplement: Translation

# Translation: Prevalência de aneurismas viscerais raros

**DOI:** 10.1590/1677-5449.202500411

**Authors:** Jamile Gomes Pereira de Barros, Luana Vieira de Medeiros Santana, Maria Clara Gomes Pontes, Letícia Lemos Belo, Bárbara Rocha Rodrigues Cox Coelho, Lucas Mendes Reis de Moura, Pedro Pereira Tenório

**Affiliations:** 1 Universidade Federal do Vale do São Francisco – UNIVASF, Paulo Afonso, BA, Brasil.

Caro Editor,

Lemos atentamente o artigo “Prevalência de aneurismas viscerais raros em 92.833 angiotomografias (2005-2021)”, o qual consideramos essencial para a comunidade acadêmica, e parabenizamos os autores pela elaboração do estudo. Entretanto, algumas fragilidades estruturais e interpretativas merecem destaque.

Ao analisarmos a proposta do estudo, observamos que os autores destacam, como único objetivo, a avaliação da prevalência de aneurismas viscerais raros. O artigo parece ter como objetivo ampliar a realização de angiotomografias, o que pode ser considerado iatrogenia na ausência de uma indicação médica plausível^1,2^. Nesse sentido, é importante avaliar de que maneira a realização de angiotomografias para o conhecimento das prevalências dos aneurismas viscerais altera desfechos clínicos para os pacientes, a fim de evitar a exposição a mais testes diagnósticos que podem não ter relevância na saúde deles^1-3^.

Os autores apontaram que 158 pacientes com aneurismas viscerais raros apresentaram 163 aneurismas, enquanto, em outro momento, mencionam 163 pacientes com aneurismas viscerais raros, o que prejudica a compreensão das informações e dos dados estatísticos apresentados.

Finalmente, congratulamos a revista e os estimados autores pela produção e publicação deste trabalho, dada a sua relevância para a comunidade científica, considerando o reduzido número de pesquisas que abordam o tema.
