# Supplementary material for: Prevalence of rare visceral aneurysms
Source: J Vasc Bras. 2025 Jul 7;24:e20250041. doi: 10.1590/1677-5449.202500412 (PMC12258811; doi:10.1590/1677-5449.202500412)
Supplement: Related texts

# Reply: Response letter

**Authors:** Sergio Quilici Belczak, Nathalia Almeida Cardoso da Silva, Matheus Toledo Nora, Paula Ribeiro do Prado Chadud, Luciana Helena Benetti, Camila de Freitas Corrêa, Adriano Tchibana, Ricardo Aun

**Affiliations:** 1 Instituto de Aprimoramento e Pesquisa em Angiorradiologia e Cirurgia Endovascular – IAPACE, São Paulo, SP, Brasil.; 2 Instituto Angiomar, São Luís, MA, Brasil.; 3 Hospital Israelita Albert Einstein, São Paulo, SP, Brasil.

Dear Readers,

Initially, we would like to thank you for your attentive comments and pertinent considerations on our manuscript “Prevalence of rare visceral aneurysms on 92,833 CAT scans (2005-2021)”, published in the Jornal Vascular Brasileiro. We are also grateful to you for valuing the subject covered and for your recognition of the study’s relevance to the scientific community.

With regard to the observations made, we present our explanations below:

**1. On the study objective:**

We understand the concerns expressed in relation to the possibility of encouraging indiscriminate use of computed tomography angiography. However, as mentioned in the article, the objective of the study was to assess the prevalence of rare visceral aneurysms in a retrospective cohort of scans conducted for a diverse range of clinical indications, not to encourage use of such examinations with no specific indication. Our intention in analyzing these data was to understand the incidence of these aneurysms in a large real-world sample from clinical practice, offering a foundation for future reflections on the magnitude of the problem. We acknowledge the importance of clarifying this point and if there is an opportunity to correct the text or publish an addendum, we would be pleased to explain in the text that by no means do we recommend indiscriminate screening, rather, we conduct analysis of a preexisting sample of scans conducted for clinical reasons.

**2. With regard to the inconsistency in the descriptions of the numbers of patients and aneurysms:**

We are grateful to you for drawing attention to the inconsistent description. After careful review, we can confirm that the correct numbers are 158 patients with rare visceral aneurysms, with a total of 163 aneurysms between them. There was in fact an error at the point where “163 patients” were mentioned, since the text should have stated “163 aneurysms”. If possible, we are also very willing to correct this point in the main text and/or in an erratum.

**3. With regard to the clinical impact of the findings and patient outcomes:**

The study objective was exclusively to conduct an epidemiological evaluation of the prevalence of rare visceral aneurysms, based on examinations conducted for a variety of reasons, without extending the analysis to the clinical outcomes related to these discoveries. We agree that the issue of the impact of these findings on clinical management is relevant, but we must emphasize that this is well beyond the scope of the study. We do, however, recognize that this is an important research gap that should be addressed in future studies, which could correlate these prevalence rates with direct clinical outcomes.

Once more, we are grateful for your careful reading and for the valuable contributions which, undoubtedly, contribute to enhancing the debate on this subject that has still received little attention in the literature.

# Reply: Carta-resposta

**Authors:** Sergio Quilici Belczak, Nathalia Almeida Cardoso da Silva, Matheus Toledo Nora, Paula Ribeiro do Prado Chadud, Luciana Helena Benetti, Camila de Freitas Corrêa, Adriano Tchibana, Ricardo Aun

**Affiliations:** 1 Instituto de Aprimoramento e Pesquisa em Angiorradiologia e Cirurgia Endovascular – IAPACE, São Paulo, SP, Brasil.; 2 Instituto Angiomar, São Luís, MA, Brasil.; 3 Hospital Israelita Albert Einstein, São Paulo, SP, Brasil.

Prezado Revisor,

Gostaríamos, primeiramente, de agradecer pelos comentários atentos e pelas considerações pertinentes ao nosso manuscrito “Prevalência de aneurismas viscerais raros em 92.833 angiotomografias (2005-2021)”, publicado no Jornal Vascular Brasileiro. Agradecemos, ainda, pela valorização do tema abordado e pelo reconhecimento da relevância deste estudo para a comunidade científica.

Com relação às observações apresentadas, seguem abaixo nossos esclarecimentos:

**1. Sobre o objetivo do estudo:**

Compreendemos a preocupação expressa quanto à possibilidade de indução à realização indiscriminada de angiotomografias. No entanto, como apresentado no artigo, o objetivo do estudo foi avaliar a prevalência de aneurismas viscerais raros em uma coorte retrospectiva de exames realizados por diversas indicações clínicas, e não estimular a realização de exames sem indicação específica. Buscamos, por meio do levantamento desses dados, compreender a ocorrência desses aneurismas em uma amostra ampla e real da prática clínica, oferecendo subsídios para reflexões futuras sobre a magnitude do problema. Reconhecemos a importância de deixar esse ponto mais claro e, caso haja oportunidade de correção ou adendo, nos colocamos à disposição para reforçar no texto que, em momento algum, se propõe a recomendação de rastreamento indiscriminado, mas sim a análise de uma amostra já formada por exames indicados clinicamente.

**2. Sobre a inconsistência na descrição do número de pacientes e aneurismas:**

Agradecemos por ter chamado atenção para a inconsistência na descrição. Após revisão atenta, confirmamos que o número correto é 158 pacientes com aneurismas viscerais raros, totalizando 163 aneurismas. Houve, de fato, um erro de digitação na parte em que se referia a “163 pacientes”, quando o correto seria “163 aneurismas”. Caso possível, também nos colocamos à disposição para corrigir esse ponto no corpo do texto e/ou em nota de errata.

**3. Sobre o impacto clínico dos achados e desfechos para o paciente:**

O presente estudo teve como foco exclusivamente a avaliação epidemiológica da prevalência dos aneurismas viscerais raros, com base em exames realizados por diferentes motivos, sem avançar para uma análise dos desfechos clínicos relacionados a essas descobertas. Concordamos com a relevância da questão levantada quanto ao impacto desses achados na conduta clínica, mas ressaltamos que esse aspecto está fora do escopo proposto para o presente trabalho. Reconhecemos, contudo, que essa é uma lacuna importante a ser abordada em estudos futuros, os quais possam correlacionar essas prevalências com desfechos clínicos diretos.

Mais uma vez, agradecemos pela leitura atenta e pelas valiosas contribuições que, sem dúvida, colaboram para o aprimoramento da discussão sobre esse tema ainda pouco explorado na literatura.
